# Urinary Metabolomics Predict Acute Kidney Injury in Very-Low-Birth-Weight Infants with Patent Ductus Arteriosus

**DOI:** 10.3390/biom16030391

**Published:** 2026-03-05

**Authors:** Moritz Niesert, Claire Cannet, Alexander Fichtner, Georg F. Hoffmann, Jürgen G. Okun, Dóra Pituk, Christian Gille, Johannes Pöschl, Sina Waldherr, Andreas Ziegler, Jens H. Westhoff

**Affiliations:** 1 Department of Pediatrics I, Center for Pediatrics and Adolescent Medicine, Medical Faculty Heidelberg, Heidelberg University, 69120 Heidelberg, Germany; 2 Bruker Biospin GmbH, 76275 Ettlingen, Germany; 3 Department of Neonatology, Center for Pediatrics and Adolescent Medicine, Medical Faculty Heidelberg, Heidelberg University, 69120 Heidelberg, Germany

**Keywords:** acute kidney injury, biomarkers, preterm infants, metabolomics, ^1^H-NMR-spectroscopy, neonatology, nephrology

## Abstract

Very preterm infants with immature kidneys exhibit high vulnerability to acute kidney injury (AKI). While AKI is associated with adverse outcomes, serum-creatinine-based diagnostics prove unreliable in early risk assessment of kidney damage. This pilot study investigated ^1^H-NMR spectroscopy-based metabolomics for the identification of very-low-birth-weight (VLBW < 1500 g) infants at risk of AKI before and during indomethacin treatment for patent ductus arteriosus (PDA). Longitudinal urine samples (0 h, 12 h, 36 h, 84 h, 120 h, 14 d, 28 d) from 12 VLBW infants receiving indomethacin for hemodynamically significant PDA were analyzed by ^1^H-NMR spectroscopy. In total, 150 urinary metabolites were annotated and single-metabolite and multivariate analyses were performed. At 36 h after treatment initiation, three patients (25%) developed AKI (KDIGO criteria). Principal component analysis (PCA) revealed significant differences in urinary metabolic profiles between the AKI and non-AKI groups 12 h after indomethacin initiation. Before treatment, five metabolites were significantly lower in the AKI group: adenine, creatine, dimethylglycine, 1-methylnicotinamide, and methylmalonic acid. Urinary creatine/creatinine (AUC 0.97) and 1-methylnicotinamide/creatinine (AUC 0.93) exhibited promising prognostic accuracy for the prediction of AKI. 1-methylnicotinamide/creatinine concentrations remained persistently reduced during the study. In conclusion, urinary metabolomics, particularly creatine and 1-methylnicotinamide levels, may serve as valuable non-invasive biomarkers for identifying VLBW infants at risk of AKI.

## 1. Introduction

Due to ongoing organogenesis, preterm infants with immature organs rank among the most vulnerable human populations with respect to detrimental external influences. While the loss of the protective environment of the uterus per se poses a burden to organ development, preterm-born infants are exposed to a variety of stresses that further impact organ development and function [1,2,3]. Since there is growing knowledge that early adverse influences on organ development can potentially affect health outcomes in later life, the prevention of organ injury is of utmost interest [2].

With incidences of up to 47.9% in very preterm neonates 22–28 weeks of gestational age [4], acute kidney injury (AKI) is very common in preterm neonates and is related to poor short-term outcomes such as prolonged invasive mechanical ventilation (IMV), length of hospital and intensive care unit (ICU) stay, and mortality [5,6]. Since more than 60% of nephrons are formed in the last trimester of pregnancy, the kidneys of very preterm infants are particularly susceptible to injury [7,8]. Risk factors, such as a hemodynamically significant PDA, nephrotoxic drugs (e.g., indomethacin), AKI and malnutrition, can impact kidney health and disease not only in the short term, but throughout the life course [9,10]. Long-term complications of neonatal AKI include proteinuria, hypertension and chronic kidney disease (CKD) [10]. Of note, intrauterine growth restriction, low birth weight and premature birth per se predispose for renal, cardiovascular and metabolic diseases in later life, rendering this population particularly vulnerable to additional nephrotoxic insults such as indomethacin-associated renal impairment [11,12,13]. Hence, risk stratification by early identification of preterm infants at high risk of AKI in combination with the implementation of mitigation and prevention strategies (e.g., cessation of nephrotoxic drug administration) is essential for short- and long-term kidney and cardiovascular outcomes [6].

While the necessity for kidney health assessment in preterm infants is high, due to a lack of appropriate diagnostic methods, the detection of imminent AKI, especially in very premature infants, remains challenging in the first days of life [6]. Serum creatinine (SCr) is a late and indirect marker of kidney function, and, due to transplacental transmission, dynamically developing glomerular filtration rates, varying degrees of creatinine reabsorption in the proximal tubule and inter-individual maturational differences, it is not a reliable gold standard in neonatal AKI diagnostics. Repeated blood sampling for the determination of the SCr concentration may lead to an increased risk of anemia in premature infants [14]. Additionally, urine output monitoring for the diagnosis of AKI is often unreliable due to the frequent absence of bladder catheters. Further, diaper weighing can be imprecise with regard to low urine volumes and evaporation. This underscores the unmet need for novel technologies for the detection of imminent AKI.

Metabolomics bears the potential to capture the intricate complexities inherent in a living system, integrating intrinsic and extrinsic factors to represent an individual’s physiological state [15]. ^1^H-nuclear magnetic resonance spectroscopy (NMR) is a widely utilized technique in metabolic profiling. NMR is non-selective and non-destructive, requires minimal sample volume and preparation efforts, allows for the analysis of intact biofluids, detects a wide range of metabolites simultaneously, and produces highly reproducible and quantifiable results, making it an efficient high-throughput analytical tool [16]. ^1^H-NMR-based metabolomics has been used to describe biological patterns and develop relevant biomarkers and prediction models in acute and chronic kidney diseases [17,18,19,20,21]. With respect to AKI in neonatal and pediatric populations, we and others have demonstrated the potential of non-invasive urine metabolite classifiers not only to detect established AKI [22], but also to identify at-risk children with high diagnostic accuracy [23]. In VLBW infants, ^1^H-NMR metabolomics has been applied to explore metabolic changes associated with prematurity, nutritional interventions, and disease states, including necrotizing enterocolitis (NEC) and bronchopulmonary dysplasia (BPD) [24]. Given these observations, NMR-based metabolomics is a promising approach to guide patient stratification in VLBW infants.

In this pilot study, we analyzed whether metabolomics by ^1^H-NMR spectroscopy is a potential tool for the identification of imminent AKI in a cohort of VLBW infants receiving indomethacin for a hemodynamically relevant PDA.

## 2. Materials and Methods

### 2.1. Study Design and Participants

Urine samples from a previously published prospective cross-sectional cohort study on VLBW preterm infants (birth weight < 1500 g) admitted to the neonatal intensive care unit of the University Children’s Hospital, Heidelberg, Germany, between March 2012 and July 2015, were analyzed [25]. In the present study, only patients who received indomethacin for a hemodynamically relevant PDA were included to minimize confounders.

Exclusion criteria were contraindications for indomethacin therapy (active bleeding, thrombocytopenia and/or coagulation defects), congenital kidney diseases, suspected or proven sepsis and suspected or proven necrotizing enterocolitis. Patients with relevant skin irritation or technical difficulties in urine collection and patients with discontinuation of indomethacin therapy were withdrawn from the study.

Indomethacin treatment was introduced in line with our institutional treatment standards of that time [25]. At postnatal age of 2–3 days, routine screening for PDA was performed by echocardiography. Criteria for indomethacin therapy in spontaneously breathing VLBW infants were PDA > 1.5 mm in diameter and left-atrium-to-aortic-root ratio > 1.4 and/or markedly reduced or retrograde diastolic flow in the anterior cerebral artery or celiac trunk with resistance index > 0.8 and/or bowing of the interatrial septum to the right with enlarged left atrium and left ventricle. In ventilated VLBW infants, every PDA > 1.5 mm at postnatal age day 2–3 was treated with indomethacin. A PDA < 1.5 mm without signs of hemodynamic relevance was not treated in VLBW infants. Following our institutional standard procedure, indomethacin was administered intravenously at three doses (0.2 mg/kg body weight per dose) at 12 h intervals. Thereafter, echocardiographic reevaluation was performed and, in case of ductal closure or ductus arteriosus diameter < 1.5 mm, two further indomethacin doses (0.2 mg/kg body weight per dose) were applied at 24 h intervals. All patients with a ductus arteriosus diameter > 1.5 mm after the third dose of indomethacin were excluded from the study. All echocardiographic measurements were performed by a board-certified consultant neonatologist with advanced training in pediatric echocardiography, as per our institution’s protocol.

For ^1^H-NMR spectroscopy, only samples that had never been thawed were included in the analysis. Three patients were excluded from the analysis due to not aligning with the criteria. The final analysis encompassed a total of 12 patients.

As this is an exploratory pilot study using untargeted metabolomics, a formal sample size calculation was not performed, as neither discriminatory features nor their effect sizes are known a priori in untargeted analyses. The sample size reflects the practical and ethical constraints of recruiting VLBW infants at a single center, and all findings are considered hypothesis-generating.

### 2.2. Sample and Data Collection

As previously described [25], following routine echocardiography, urine and blood specimens were collected immediately before (0 h), during (6, 12, and 36 h), and after (84 and 120 h, days 7, 14, and 28) indomethacin administration according to standard procedure. Neonatal AKI was diagnosed according to the neonatal modified Kidney Disease Improving Global Outcomes (KDIGO) criteria [26]. Then, 12 h urine collections for determination of creatinine clearance were obtained at 12–24 and 84–96 h following study enrollment. Creatinine clearance (CLcr) was calculated as: CLcr (mL/min/1.73 m^2^) = (Creatinine in urine/Creatinine in serum) × (Volume of urine/Time) × 1.73.

Urine was obtained using adhesive urinary bags (U-Bag^®^ Sterile Cloth Adhesive Premature Size, Briggs Healthcare™, Moines, IA, USA) in accordance with standard procedural recommendations. Serum and urinary creatinine, urea, cystatin C, and protein determinations were performed in the institution’s accredited clinical routine laboratory. Urine samples for ^1^H-NMR spectroscopy measurements were centrifuged, the supernatant was stored in aliquots at −80 °C and thawed immediately prior to analysis.

### 2.3. ^1^H-NMR-Spectroscopy

Samples were analyzed under full automation using a Bruker Avance IVDr platform (600 MHz Avance NEO, Bruker Biospin GmbH, Ettlingen, Germany), equipped with a SampleJet sample changer with sample cooling and a pre-heating station. The system was equipped with a 5 mm inverse probe with z-gradient, automated tuning and matching, and a BCU-I cooling unit. Data acquisition and processing were conducted using Bruker’s body fluid NMR method package B.I. Methods 2.5, controlled by ICON NMR (Bruker Biospin GmbH, Ettlingen, Germany).

Prior to analysis, samples were equilibrated for 5 min within the NMR probe head at 27 °C (300 K), automated procedures were employed for tuning and matching, locking, shimming, lock phase optimization, and hard pulse calibration (90 °C) to ensure optimal experimental conditions for NMR. One-dimensional ^1^H-NMR NOESY (Nuclear Overhauser Effect SpectroscopY, 32-scan) spectra were acquired using a standard pulse sequence with water signal suppression (Bruker pulse program library noesygppr1d, Bruker Biospin GmbH, Ettlingen, Germany). Subsequent data processing, including Fourier transformation, automated phasing, and baseline correction, was performed using the Bruker standard automation program APK0.NOE (Bruker Biospin GmbH, Ettlingen, Germany). Quantitative calibration of the spectra was achieved via the PULCON principle, utilizing an external reference sample (QuantRefC Calibrator, Bruker Biospin GmbH, Ettlingen, Germany). At least 350 µL of urine was analyzed per sample. Trimethylsilyl propionic acid-d4 sodium salt (TSP) was used as the reference peak for chemical shift calibration.

### 2.4. Spectral Binning

For creating the bucket table, spectral intensity was scaled using a minimum baseline scale using the 1.0–4.3 ppm region to eliminate any variability coming from spot urine. Afterward, each spectrum was segmented from 0.8 to 9 ppm into consecutive fixed bins containing 0.01 ppm, and the pertaining regional integrals (bin intensities) excluding the 4.5–6.5 ppm region were calculated.

### 2.5. Statistical Analysis

Data is presented as median and minimum to maximum due to small sample sizes and the assumed non-normal distribution. Fisher’s exact test (for categorical) and non-parametric Mann–Whitney U-test (for continuous variables) were used. Receiver operating curves (ROC) were calculated to evaluate the diagnostic accuracy of selected metabolites. *p* values < 0.05 were considered significant.

Concentrations of 150 urinary metabolites were obtained by Bruker IVDr Quantification in urine for children module (B.I.QuantUR-e 1.1, Bruker Biospin GmbH, Ettlingen, Germany), and metabolite concentrations below the detection limit were considered to be zero. Metabolite concentrations were normalized and stated as mmol/mol creatinine to minimize effects from the inherent variability in spot urine. Concentrations of each metabolite were scaled first by mean centering and then division by standard deviation (auto-scaling) prior to multivariate analysis.

For volcano plot analysis, a change of more than or equal to 1.5-fold was considered biologically significant, and a false discovery rate (FDR) corrected *p*-value < 0.05 was considered statistically significant.

Cliff’s delta (δ) was used as a non-parametric effect size measure. Effect sizes were interpreted as negligible (δ < 0.147), small (0.147 < δ < 0.33), medium (0.33 < δ < 0.474), or large (δ > 0.474).

Principal component analysis (PCA), permutational analysis of variance (PERMANOVA) with 999-fold permutation, fold-change analysis (FC) and ROC analysis were performed using MetaboAnalyst 6.0 (Xia Lab, Montreal, QC, Canada) and R (Version 4.3.1, The R-Foundation, Vienna, Austria) [27,28]. Metabolites with constant values across all samples were excluded from PCA. Metabolite fold changes between the groups were assessed using the Mann–Whitney U-test. Optimal cutoff values for ROC analysis were determined by the Youden method [29]. All other statistical analyses were performed using IBM SPSS (Version 29.0, Armonk, NY, USA) and R (Version 4.3.1, The R-Foundation, Vienna, Austria).

## 3. Results

### 3.1. Characteristics of Study Cohort

A total of 12 VLBW infants receiving indomethacin treatment for PDA were included in the study; no patient discontinued therapy prematurely. Three out of twelve patients were included on the third postnatal day (AKI group 2/3 vs. 1/9 non-AKI group, *p* = 0.06), and all other patients were enrolled on the second postnatal day. Then, 36 h (h) after study enrollment, three (25%) indomethacin-exposed VLBW infants developed AKI according to neonatal KDIGO criteria (AKI group). All three patients fulfilled SCr, but not the urine output KDIGO AKI criteria. Baseline characteristics of VLBW infants in the non-AKI and AKI groups showed no significant differences in patient demographics, medical interventions, nutritional parameters, prenatal risk factors for kidney injury, respiratory status, or laboratory values, except for significantly lower serum creatinine (SCr) (AKI group: 0.66 mg/dL vs. non-AKI group: 0.87 mg/dL, *p* < 0.05) and urea (AKI group: 31 mg/dL vs. non-AKI group: 66 mg/dL, *p* < 0.05) in the AKI group. Details are shown in Table 1.

### 3.2. Traditional Parameters of Kidney Function and Injury

As demonstrated before [25], SCr concentrations remained persistently elevated until day 5 (48 h after the last indomethacin dose). SCr then declined over the following 48 h. Additionally, 36 h after treatment initiation, three (25%) indomethacin-exposed VLBW infants developed AKI as defined by the neonatal KDIGO AKI criteria (stage 1, n = 2; stage 2, n = 1). Of note, all three patients fulfilled SCr, but only one patient also fulfilled the urine output KDIGO AKI criteria (SCr AKI stage 2; urine output AKI stage 1).

### 3.3. Urinary Metabolomics Analysis

#### 3.3.1. Fold-Change Analysis

**Figure 1:**
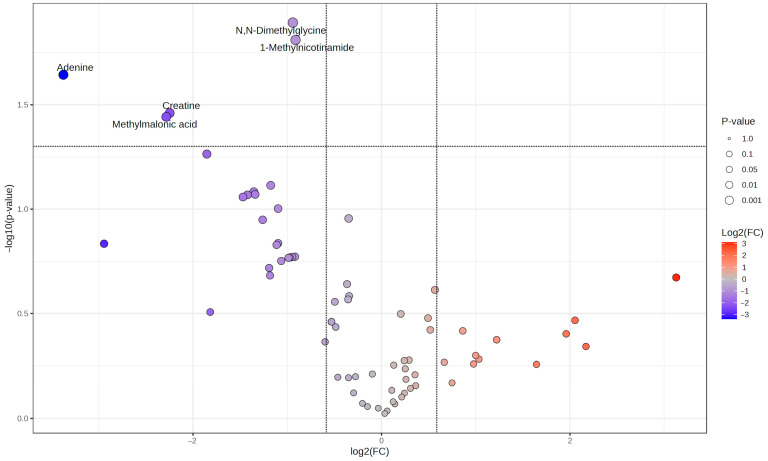
Alterations in metabolite concentrations between AKI and non-AKI groups prior to treatment initiation with indomethacin (0 h). Volcano plot showing differential abundance of urinary metabolite concentrations between groups. *X*-axis: log2(FC), *Y*-axis: −log10 (fdr-adjusted *p*-value). Thresholds for significance were defined as follows: a fold change > 1.5 was applied as a commonly used criterion in untargeted metabolomics to emphasize biologically meaningful differences and to limit the influence of small effect sizes that may arise from feature-level analytical variability and signal uncertainty inherent to untargeted ^1^H-NMR spectroscopy data (vertical dotted line, log_2_(1.5) = 0.585), while an FDR-adjusted *p*-value < 0.05 indicated statistical significance (horizontal dotted line, −log_10_(0.05) = 1.301) Red points indicate metabolites increased in AKI patients; blue points show decreased metabolites, with point size corresponding to statistical significance. Five metabolites showed alterations above FC and *p*-value threshold: Adenine, creatine, methylmalonic acid, N,N-dimethylglycine and 1-methylnicotinamide. AKI: acute kidney injury, FC: fold change.

In total, 150 urinary metabolite concentrations were included in the analysis. As shown in Figure 1, which displays a volcano plot comparing the two groups, the original concentrations of five urinary metabolites were significantly reduced in the AKI group compared to the non-AKI group, both biologically (fold change (FC) > 1.5) and statistically (*p* < 0.05): Adenine (FC = 10.38, *p* = 0.03), creatine (FC = 4.75, *p* = 0.04), dimethylglycine (FC = 1.92, *p* = 0.01), 1-methylnicotinamide (FC = 1.88, *p* = 0.04) and methylmalonic acid (FC = 4.87, *p* = 0.04). Metabolite concentrations were normalized and auto-scaled for fold-change analysis and PCA. The following creatinine-normalized unscaled concentrations were observed in the AKI and the non-AKI groups (median (*p*-value)): adenine: 0.0 vs. 20.0 mmol/mol creatinine (*p* = 0.03); creatine: 69.2 vs. 350.0 mmol/mol creatinine (*p* = 0.04); dimethylglycine: 46.2 vs. 71.4 mmol/mol creatinine (*p* = 0.06); 1-methylnicotinamide 12.1 vs. 218.2 mmol/mol creatinine (*p* = 0.04); methylmalonic acid: 0 vs. 14.3 mmol/mol creatinine (*p* = 0.15). Excretion of adenine and methylmalonic acid was below the detection limit in all AKI patients. Differences in the median urinary dimethylglycine concentration did not reach statistical significance; therefore, these metabolites were excluded from the subsequent ROC analysis.

#### 3.3.2. Pre-Treatment AKI Group Membership Does Not Explain Metabolomic Profile

**Figure 2:**
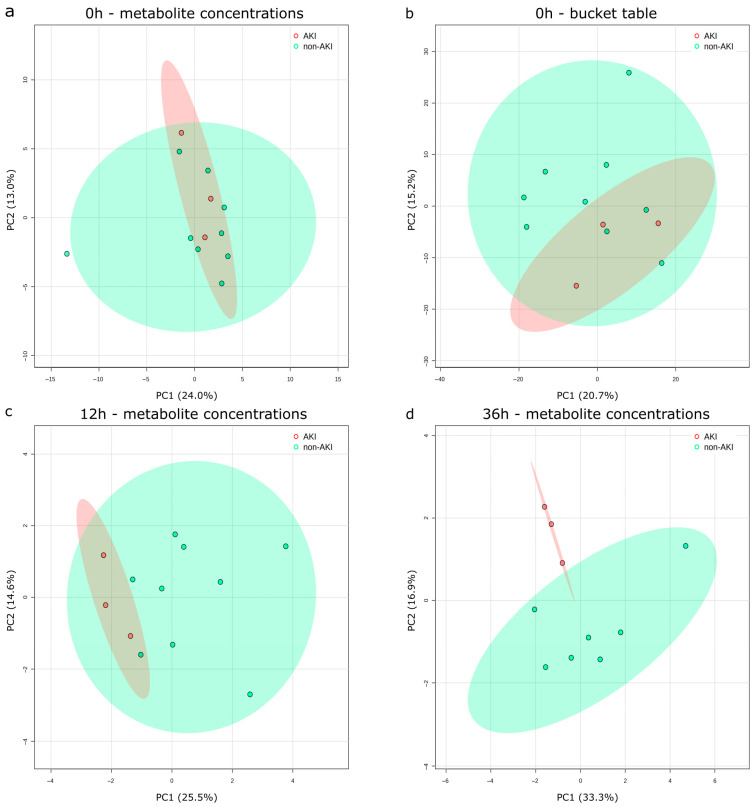
Principal component analysis of urinary metabolic profiles in AKI and non-AKI VLBW infants. At baseline, prior to indomethacin treatment, PCA showed no distinctive separation between groups, regardless of whether metabolite concentrations (**a**) or buckets (**b**) were used. 12 h after treatment initiation (**c**), PCA showed an enhanced separation between groups, with even more pronounced separation 36 h after indomethacin treatment initiation (**d**). PC1 and PC2, with respective variance percentages, are displayed on each axis; 95% confidence interval ellipses outline each group (red: AKI group, green: non-AKI group). PCA: principal component analysis, PC 1/2: 1st/2nd principal component.

PCA on individual metabolite concentrations (Figure 2a) revealed that the first two principal components (PC) explained 37% of the total variance (PC1: 24%, PC2: 13%). Subsequently, the performed PERMANOVA analysis showed no significant differences between the AKI and the non-AKI groups (F = 0.53, R^2^ = 0.50, *p* = 0.6), but should be interpreted with caution due to the small sample size. Furthermore, we performed a PCA on the bucket table (Figure 2b) to include information from non-annotated segments of the spectrum, showing similar results; 35.9% of total variance was explained by the first two components (PC1: 20.7%, PC2: 15.2%). PERMANOVA showed no significant differences between the groups (F = 1.10, R^2^ = 0.10, *p* = 0.36).

#### 3.3.3. Urinary Metabolomic Profiles Detect AKI Prior to Neonatal KDIGO-Based Criteria

In order to evaluate the longitudinal development of differences between the groups, a PCA was conducted, followed by a PERMANOVA analysis at 12 h and 36 h after indomethacin initiation. Then, 12 h after treatment initiation, PC1 and PC2 accounted for 40.1% (see Figure 2c, PC1: 25.5%, PC2: 14.6%) of the overall variance, and PERMANOVA revealed significant differences between the groups (F = 3.35, R2 = 0.25, *p* = 0.049, 999 permutations). Additionally, 36 h after treatment initiation with indomethacin, all three AKI patients fulfilled neonatal KDIGO criteria. At this time point, 50.2% of the total variance was explained by the first two components (see Figure 2d, PC1 33.3%, PC2 16.9%) and groups showed a significant difference explaining 33% of the observed variance (F = 3.86, R2 = 0.33, *p* = 0.03). Given the limited sample size within the study groups, the generation of supervised multivariate models was not feasible, as this would have led to significant overfitting.

#### 3.3.4. Prognostic Accuracy of 1-Methylnicotinamide/Creatinine and Creatine/Creatinine in Predicting AKI

**Figure 3:**
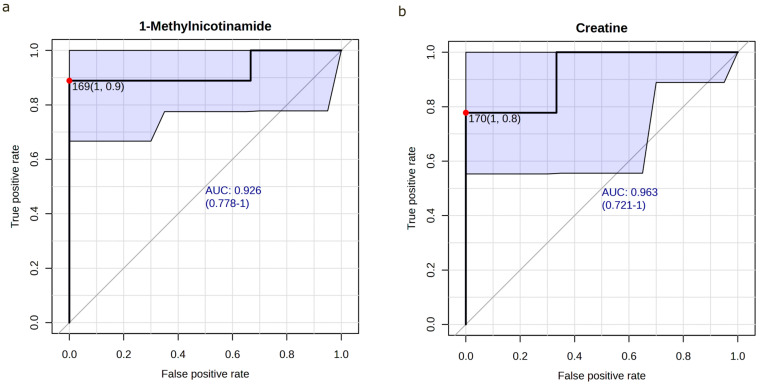
ROC analysis of 1-methylnicotinamide and creatine prior to indomethacin treatment. Receiver operating characteristic (ROC) curve evaluating the diagnostic performance of: (**a**) 1-methylnicotinamide/creatinine with AUC 0.926 (95% CI 0.78–1.00). (**b**) creatine/creatinine with AUC 0.96 (95% CI 0.72–1.00). Optimal cutoff was determined by the Youden method. AUC: area under the curve, CI: confidence interval.

Urinary 1-methylnicotinamide and creatine were selected for further evaluation as potential prognostic markers regarding the development of AKI in patients undergoing indomethacin therapy. In ROC analysis, creatine/creatinine (AUC 0.97 [0.70–1.00], cutoff 170 mmol/mol creatinine) and 1-methylnicotinamide/creatinine (AUC 0.93 [0.67–1.00], cutoff: 169 mmol/mol creatinine) showed an excellent separation between the AKI and non-AKI groups prior to treatment as well as 12 h after treatment initiation (creatine/creatinine AUC 0.93 [0.70–1.00], cutoff: 170 mmol/mol creatinine, 1-methylnicotinamide/creatinine AUC 0.93 [0.78–1.00], cutoff: 169 mmol/mol creatinine) (Figure 3). Thus, reduced 1-methylnicotinamide and creatine excretion prior to and 12 h after treatment initiation is associated with indomethacin-induced AKI in VLBW infants. Cliff’s delta was −0.85 (95% CI [−0.98, −0.24]) for creatine/creatinine and −0.85 (95% CI [−0.99, 0.20]) for 1-methylnicotinamide/creatinine, both indicating large effect sizes. In 25 of 27 pairwise comparisons (93%), both metabolite concentrations were lower in the AKI group.

#### 3.3.5. Longitudinal Analysis of Urinary Creatine and 1-Methylnicotinamide

A longitudinal analysis was conducted to further examine the temporal alterations in urinary 1-methylnicotinamide and creatine levels. Urinary creatine levels fell below the detection limit in the AKI group at 12 h and 36 h. Furthermore, a significant decrease (*p* < 0.05) in creatine levels was observed in the AKI group at 0 h, 12 h, and 36 h compared to the non-AKI group. 1-methylnicotinaminde concentrations showed equal dynamics, with significantly (*p* < 0.05) lower urinary concentrations at 0 h and 12 h after treatment initiation and again at days 14 and 28. Detailed results for urinary creatine/creatinine and 1-methylnicotinamide/creatinine concentrations are demonstrated in Table 2.

## 4. Discussion

Metabolomics by ^1^H-NMR spectroscopy is a powerful and non-invasive tool with the capacity to comprehensively assess the state of an individual. However, due to the untargeted approach and a myriad of data included for each subject, a close examination of relevant confounders is essential. As the urinary metabolome (and proteome) in term and preterm infants is influenced by gestational age, birth weight, nutrition, nephrotoxic medication and the presence of a PDA [30,31], a rigorous control group of patients receiving the same nephrotoxic medication and reflecting the other potential major confounders is required [32]. The AKI group resembled the overall cohort regarding gestational age, birth weight, sex ratio, medical intervention, nutritional factors and cumulative indomethacin dose. Traditional markers of kidney function were equal or lower (SCr and urea) in the AKI group prior to indomethacin treatment. In all three patients in the AKI group, AKI according to the neonatal KDIGO criteria was detected 36 h after treatment initiation.

PCA of pre-treatment samples showed consistent results across both annotated metabolite concentration and bucket table datasets, with the majority of variance attributed to annotated spectral regions. Initially, no statistically significant between-group differences in metabolic profiles were detected, regardless of whether non-annotated spectral regions were included. This similarity of metabolic profiles between groups likely reflects the effective control of relevant confounding factors in our cohort. However, longitudinally repeated PCA analyses showed an increasing portion of variance explained by the AKI group membership. These findings reached statistical significance. In accordance with the findings of preceding publications [20,23], statistically significant discrepancies in urinary metabolic profiles were observed between the AKI and non-AKI groups as early as 12 h following the commencement of nephrotoxic indomethacin treatment, i.e., 24 h prior to the fulfillment of neonatal KDIGO AKI criteria. This temporal advantage emphasizes the potential of urinary metabolomics for early detection of imminent AKI, prior to the fulfillment of KDIGO AKI criteria including SCr and urine output. It is evident that this temporal advantage has the potential to establish a window for early therapeutic interventions.

However, even more urgent than early detection is the identification of patients at risk of developing AKI before the commencement of nephrotoxic treatment. In VLBW infants, defining high-risk patients prior to indomethacin treatment bears the potential to identify those at the highest risk, protocolize AKI surveillance, improve prevention and diagnosis, and expand kidney support therapy. PCA prior to treatment initiation showed no significant differences between groups. Supervised multivariate models are susceptible to overfitting and inappropriate due to the low sample size. Hence, an univariate metabolite analysis was carried out.

The fold-change analysis and t-tests revealed five metabolites significantly altered between the groups prior to treatment initiation. Creatine/creatinine and 1-methylnicotinamide/creatinine ratios were selected for further characterization as described. Both metabolites showed a promising predictive value (ROC-AUC = 0.9) for the prediction of imminent AKI prior to initiation of indomethacin therapy. Creatine/creatinine showed no significant differences in the longitudinal analysis after 36 h, while 1-methylnicotinamide/creatinine was significantly altered 14 days and 28 days after treatment initiation. Reduced urinary 1-methylnicotinamide and creatine concentrations have been shown to be associated with prematurity and low birth weight [32,33,34]. Both confounders showed no significant difference between groups. As the creatine/creatinine ratio is higher in term than in preterm neonates [35], a reduced creatine/creatinine ratio may be indicative of an individual immaturity of the neonatal kidney, which renders it more vulnerable to exogenous noxae. Notably, nephrogenesis in humans is not complete until approximately 36 weeks of gestational age, meaning that the kidneys of VLBW infants are still undergoing active development with incomplete nephron endowment, immature tubular transport systems, and limited functional reserve. A reduced creatine/creatinine ratio may thus not merely reflect differences in filtration but rather represent an individual degree of renal developmental immaturity, identifying neonates whose kidneys are particularly susceptible to nephrotoxic insult due to their limited capacity to compensate for pharmacologically induced reductions in renal perfusion.1-methylnicotinamide is a metabolite of the nicotinamide pathway, a metabolic hub for various metabolic pathways in renal tubular epithelium [36]. Mitochondrial function and redox homeostasis, cellular energy metabolism, genomic stability, gene expression, processing of RNA, and inflammation are some of the various physiological functions linked to the nicotinamide pathway [36]. The kidney is one of the organs with the highest cellular levels of metabolites of the nicotinamide pathway [37] and the NAD+ metabolism seems to play a crucial role in kidney development [38].

In this pilot study, even before the initiation of indomethacin treatment (at timepoint 0 h), urinary 1-methylnicotinamide levels were found to be statistically and biologically significantly reduced in VLBW infants who later developed AKI according to the neonatal KDIGO criteria. As demonstrated by Poyan Mehr et al., alterations in nicotinamide pathways have been associated with an increased risk of AKI in both animal models and human patients [39]. These findings suggest the presence of a potential subgroup of VLBW infants who exhibit an impaired nicotinamide pathway function and, consequently, a heightened risk of adverse nephrological outcomes. Reduced urinary 1-methylnicotinamide levels prior to indomethacin exposure may therefore indicate a developmental impairment of NAD^+^ metabolism in these infants, rendering their kidneys particularly vulnerable to additional nephrotoxic insults, explaining the heightened AKI susceptibility in this subgroup.

Furthermore, various clinical trials assessing the protective and therapeutic value of NAD+-precursors are currently under investigation [40,41]. A translation in neonates might be a promising future approach. It is interesting to note that the levels of nicotinamide in the urine remain lower than those in the non-AKI group over the entire duration of the study.

In addition to the aforementioned potential alterations in the NAD+ metabolism, reduced 1-methylnicotinamide has also been proposed as a sensitive marker of renal secretory function for organic cations (e.g., creatine) and renal blood flow [42,43]. Since indomethacin reduces renal blood flow [44], patients in the AKI group who excrete less 1-methylnicotinamide may be particularly susceptible to a further decrease. It is hypothesized that alterations to the nicotinamide pathway, reduced renal blood flow, or both, put preterm infants at risk of AKI when exposed to nephrotoxic medication.

Potential nephrotoxic damage in VLBW infants is not limited to indomethacin alone but rather multifactorial, as VLBW infants are regularly exposed to a variety of renal risk factors. These include the hemodynamic relevance of the ductus arteriosus per se, nephrotoxic co-medication such as the aminoglycoside gentamicin, infection/sepsis, invasive mechanical ventilation, differences in the APGAR score, perinatal asphyxia, necrotizing enterocolits and others. Of note, the results of our study do not allow any conclusions regarding the main underlying etiology of AKI, as there were no relevant differences in the investigated pre-, peri-, and postnatal factors between the non-AKI group and the AKI group. In this context, a pre-existing impairment of the nicotinamide pathway or renal developmental immaturity, as suggested by the findings of this study, may lower the threshold at which these cumulative insults result in clinically manifest AKI.

It is important to acknowledge several limitations of the present study. The most significant of these is the limited sample size, which is inherently linked to the exploratory, hypothesis-generating character of the study. In metabolomic studies, especially in small cohorts, the biological plausibility of identified metabolites is of particular significance. The findings of the present study are in accordance with those of previous studies [31,32,35,36,37,38,39,40] and are biologically plausible; both metabolite concentrations exhibit large, concordant effects (δ = −0.85). In view of the limited number of subjects within the present study, the employment of supervised classification models for feature selection was eschewed. The employment of such models in future, larger cohorts has the potential to enhance the scientific yield. Consequently, independent, confirmatory studies with a larger number of patients are required to validate our findings. Furthermore, our findings underscore the ongoing need to identify and validate reliable blood- and urine-based AKI biomarkers in neonates, as currently available markers remain insufficient for timely detection and risk prediction in VLBW infants. However, given the challenges associated with sample collection in VLBW infants, we consider these findings to be a valuable starting point for subsequent research projects.

Due to technical limitations, the absolute detection limit is low in ^1^H-NMR spectroscopy, and it was not possible to obtain exaggerated information on most of the nicotinamide pathway. Consequently, there is a clear necessity for further studies that employ alternative methods, for example, targeted liquid chromatography–tandem mass-spectrometry-based metabolomics (LC-MS MS). These studies will provide a more comprehensive overview of the biological processes involved in the nicotinamide pathway in premature infants. Combined with serial blood flow measurements, these studies have the potential to further clarify the mechanisms that increase the risk of AKI in VLBW infants.

## 5. Conclusions

In conclusion, in this pilot study, urinary metabolomics by ^1^H-NMR spectroscopy were able to identify patients at risk of AKI before the initiation of indomethacin treatment for a hemodynamically relevant PDA. Urinary creatine and 1-methylnicotinamide, standardized to creatinine, have been identified as promising markers to detect VLBW infants at risk of developing AKI prior to indomethacin for hemodynamically significant PDA.

Despite its limited sample size, our present pilot study demonstrates that ^1^H-NMR-based metabolomics has the potential to identify VLBW infants who are at risk of developing indomethacin-associated renal impairment. The non-invasive nature of urinary metabolomics suggests that this method may be of great benefit for monitoring and guidance, i.e., in this very vulnerable patient population. Further research is necessary to substantiate these findings, elucidate the role of nicotinamide in AKI risk in VLBW infants, and evaluate the ability of this approach to distinguish reversible from irreversible kidney injury in complex neonatal conditions, such as severe perinatal asphyxia, sepsis, or Twin-to-Twin Transfusion Syndrome, enhancing its clinical applicability.

## Figures and Tables

**Table 1:** Baseline characteristics of study cohort.

	Non-AKI Group	AKI Group	Significance
**Patient Demographics**			
n	9	3	-
Gestational age (weeks + days)	26 + 6 (24 + 0–30 + 0)	28 + 4 (27 + 6–29 + 5)	0.37
Birth weight (g)	1100 (650–1480)	1100 (750–1430)	0.89
Birth weight (SDS)	0.39 (−0.59–0.80)	−0.23 (−1.33–0.34)	0.15
Male:Female	7:2	3:0	-
Umbilical cord arterial blood pH	7.32 (7.10–7.43)	7.25 (7.23–7.32)	0.21
APGAR 1 min			0.57
3	2 (22.2%)	0 (0%)	
5	2 (22.2%)	0 (0%)	
6	1 (11.2%)	0 (0%)	
7	1 (11.1%)	1 (33.3%)	
8	3 (33.3%)	2 (66.6%)	
APGAR 5 min			0.51
5	2 (22.2%)	0 (0%)	
7	3 (33.3%)	0 (0%)	
8	1 (11.1%)	1 (33.3%)	
9	3 (33.3%)	2 (66.6%)	
APGAR 10 min			0.51
7	3 (33.3%)	0 (0%)	
8	2 (22.2%)	1 (33.3%)	
9	4 (44.4%)	2 (66.6%)	
Minimum urine output 12 h before treatment [mL/kg/d]	5.04 (1.86–8.33)	4.16 (3.09–7.38)	>0.99
Intraventricular hemorrhage (all 1st degree)	1 (11.1%)	1 (33.3%)	0.46
**Respiratory distress syndrome**	8 (88.9%)	2 (66.7%)	0.58
**Presence of proven sepsis**	0/9 (0%)	0/3 (0%)	-
**Laboratory values**			
Serum creatinine [mg/dL]	0.86 (0.53–1.36)	0.66 (0.54–0.78)	*0.04*
Serum urea [mg/dL]	66 (30–95)	31 (19–44)	*0.02*
Serum cystatin C [mg/L]	1.42 (0.61–2.62)	1.31 (1.04–1.33)	0.86
Urinary creatinine [mg/dL]	9.19 (4.17–30.83)	10.35 (6.49–14.26)	0.38
Urinary protein/creatinine [g/mol]	206.27 (83.79–635.57)	156.28 (75.41–188.25)	0.14
Urinary beta-2-microglobulin/creatinine [g/g]	9.77 (2.92–55.10)	9.34 (5.07–14.28)	0.38
**Medical intervention**			
Fluid supply on study enrollment (mL/kg per day)	110 (65–150)	110 (90–110)	0.86
Ampicillin treatment	8 (88.9%)	3 (100%)	>0.99
Cefotaxime treatment	3 (33.3%)	0 (0%)	0.49
Cefuroxime treatment	1 (11.1%)	0 (0%)	>0.99
Furosemide treatment	1 (11.1%)	0 (0%)	>0.99
Gentamicin treatment	7 (77.8%)	3 (100%)	>0.99
Hydrochlorthiazide treatment	3 (33.3%)	0 (0%)	0.51
Hydrocortisone treatment	6 (66.7%)	0 (0%)	0.18
Meropeneme treatment	0 (0%)	0 (0%)	-
Teicoplanin treatment	2 (22.2%)	0 (0%)	>0.99
Umbilical artery catheter placement	6 (66.7%)	0 (0%)	0.18
Umbilical vein catheter placement	6 (66.7%)	0 (0%)	0.18
Invasive mechanical ventilation	7 (77.8%)	1 (33.3%)	0.23
Mean airway pressure [cmH_2_O]	7.00 (6.00–8.20)	7.10 (7.10–7.10)	0.95
Fraction of inspired oxygen (FiO_2_)	0.25 (0.21–0.56)	0.21 (0.21–0.21)	0.31
Non-invasive respiratory support (CPAP)	2 (22.2%)	1 (33.3%)	0.78
Positive end-expiratory pressure [cmH_2_O]	4.85 (4.70–5.00)	5.00 (5.00–5.00)	0.67
Fraction of inspired oxygen (FiO_2_)	0.21 (0.21–0.21)	0.21 (0.21–0.21)	-
**Nutrition**			
Parenteral nutrition	9 (100%)	3 (100%)	-
Glucose [g/kg bodyweight/d]	6.90 (5.70–8.90)	8.40 (8.40–8.50)	0.06
Amino Acids [g/kg bodyweight/d]	1.50 (1.00–2.50)	2.00 (1.50–2.00)	0.37
Lipids [g/kg bodyweight/d]	0	0	-
Enteral nutrition			
Maltodextrin (15% or 25%)	3 (100%)	9 (100%)	-
**Prenatal factors**			
Amniotic infection	1 (11.1%)	0 (0%)	>0.99
HELLP	0 (0%)	0 (0%)	-
In vitro fertilization	1 (11.1%)	0 (0%)	>0.99
Intrauterine growth retardation	0 (0%)	1 (33.3%)	0.25
Maternal arterial hypertension	0 (0%)	0 (0%)	-
Maternal diabetes mellitus	0 (0%)	0 (0%)	-
Maternal use of nephrotoxic medication	0 (0%)	0 (0%)	-
Preeclampsia	0 (0%)	0 (0%)	-
Premature rupture of membranes	2 (22.2%)	0 (0%)	0.47
Twin	5 (55.6%)	1 (33.3%)	>0.99

Patient demographic and clinical characteristics were evaluated at baseline (0 h) before indomethacin administration. Data are presented as absolute values, ratios, or as median (minimum-maximum) values due to the limited sample size. Between-group comparisons were performed using the non-parametric Mann–Whitney U-test for continuous variables. Statistical significance was defined as *p* < 0.05.

**Table 2:** Longitudinal concentrations of urinary creatine and 1-methylnicotinamide.

	Non-AKI Group	AKI Group	Significance
**1-Methylnicotinamide (mmol/mol crea)**			
0 h	218.18	123.08	*0.04*
12 h	176.92	94.12	*0.02*
36 h	144.44	82.35	*0.07*
84 h	180.00	77.60	0.11
120 h	177.78	80.42	0.33
14 d	200.00	80.00	*0.02*
28 d	200.00	81.81	*0.02*
**Creatine (mmol/mol crea)**			
0 h	350.00	69.23	*0.04*
12 h	200.00	0.00	*0.02*
36 h	225.00	0.00	*0.02*
84 h	66.67	31.81	0.46
120 h	66.67	43.75	1.00
14 d	88.89	0.00	0.48
28 d	58.33	0.00	0.63

Longitudinal concentrations of 1-methylnicotinamide and creatine. Concentrations are reported as median and standardized for creatinine concentrations. Between-group comparisons were performed using the non-parametric Mann–Whitney U-test for continuous variables. Statistical significance was defined as *p* < 0.05. AKI: Acute kidney injury.

## Data Availability

To protect patient data in our vulnerable group, clinical and spectroscopy data are only available upon justified request to the corresponding author. Biomaterials are available upon justified request to the corresponding author for other research projects.

## Abbreviations

The following abbreviations are used in this manuscript:

AKI	Acute kidney injury
AUC	Area under the curve
BPD	Bronchopulmonary dysplasia
CKD	Chronic kidney disease
FC	Fold change
FDR	False detection rate
ICU	Intensive care unit
IMV	Invasive mechanical ventilation
KDIGO	Kidney Disease: Improving Global Outcomes
LC-MS	Liquid chromatography-mass spectrometry
NEC	Necrotizing enterocolitis
NMR	Nuclear magnetic resonance
NOESY	Nuclear overhauser effect spectroscopy
PC	Principal component
PCA	Principal component analysis
PDA	Persistent *ductus arteriosus botalli*
PERMANOVA	Permutational multivariate analysis of variance
ROC	Receiver operating characteristic
SCr	Serum creatinine
VLBW	Very low birthweight
